# Machine Learning Phase Classification of Thermoelectric Materials

**DOI:** 10.3390/ma18204726

**Published:** 2025-10-15

**Authors:** Chung T. Ma, S. Joseph Poon

**Affiliations:** 1 Department of Physics, University of Virginia, Charlottesville, VA 22904, USA; sjp9x@virginia.edu; 2 Department of Material Science and Engineering, University of Virginia, Charlottesville, VA 22904, USA

**Keywords:** thermoelectric, machine learning, phase classification

## Abstract

In this study, we employ a Support Vector Machine (SVM) model to efficiently classify the phases of thermoelectric (TE) alloys. While ab initio calculations and experiments have explored the phases of functional TE materials, the large variety of alloys makes these explorations time-consuming and expensive. Therefore, there is a critical need for time-efficient methods to accelerate the discovery and development of new TE materials. Recently, machine learning (ML) classification models have been applied to predict material phases, including those of multi-principal element alloys. Using an SVM to classify phases of TE alloys, our results demonstrate that the model achieves prediction accuracies ranging from 77% to 92%. Additionally, cross-validation across various TE phases is performed to demonstrate the model’s robustness in phase differentiation. This work offers a time-efficient computational approach to distinguish TE material phases, offering valuable insights that can aid in the evaluation and design of high-performance thermoelectric materials.

## 1. Introduction

Conventional energy generation loses a considerable amount of energy to waste heat [1]. Transforming this waste heat into electricity will significantly advance the efficiency of energy production. Thermoelectric (TE) technology has the goal of turning this waste heat into electricity [2,3,4,5]. In addition, thermoelectric materials have been investigated for improving battery thermal management, which can optimize the efficiency and lifespan of these systems [6,7]. A wide range of materials has been investigated for thermoelectric applications, including Half-Heusler (HH) compounds [8,9,10,11], $Bi_{2}$$Te_{3}$-based alloys [12,13,14], Ge, Pb, transition metal (TM) chalcogenides [15,16,17,18,19,20,21,22,23], oxides [24,25,26], and $Mg_{2}$(Si or Sb)-based alloys [27,28,29]. The phase formations of these materials can greatly affect their thermoelectric properties. In this context, phase formation refers to the development of specific crystal structures. For instance, in Half-Heusler (HH) compounds, it corresponds to the formation of the XYZ face-centered cubic (FCC) structure [9]. Many ab initio calculations and experiments have investigated phase formations in thermoelectric alloys. However, with many types of thermoelectric materials to choose from, identifying and differentiating their phases can be time-consuming and costly.

To this end, recent studies have employed machine learning (ML) models as an efficient method to identify phase formation in various compositionally complex or high-entropy alloys, correspondingly known as CCAs or HEAs. These phases include B2, face-centered cubic (FCC), body-centered cubic (BCC), hexagonal, and amorphous. In these studies, databases of materials have been constructed using both experimental data and first-principles calculations [30,31,32,33,34,35,36,37,38]. Both elemental parameters and alloy parameters have been used as descriptors in these models. Feature selection techniques [31,32,33,39], including correlation coefficients and wrapper methods, have been applied to select relevant raw features. Furthermore, feature engineering methods, such as using math variations and one-hot encoding, have been employed to enhance the model’s performance. Different kinds of classification models [30,31,32,33,34,35,36,37,38], including Support Vector Machine (SVM), random forest (RF), neural network (NN), and gradient boosting machine (GBM), have been developed. These models have been used to categorize various types of alloys, including multi-principal element alloys and high-entropy alloys. Some of these models have shown excellent predictive ability, achieving an accuracy of over 90%. Furthermore, regression models have predicted numerous thermoelectric properties, including figure of merit ZT, Seebeck Coefficient S, and thermal conductivity κ [40,41,42,43,44,45,46]. Some of these models have achieved a coefficient of determination, $R^{2}$, of over 0.90. In addition, the application of machine learning to a complex and diverse material system parallels approaches in other fields, for example, the use of physical parameters to optimize traffic flow dynamics [47].

While several studies have employed classification models to investigate phase formation in complex alloys, and regression models to predict thermoelectric properties, there is a noticeable lack of research specifically focused on the phase classification of thermoelectric materials. As previously discussed, the phase of a thermoelectric material plays a critical role in determining its thermoelectric performance. Therefore, developing a time-efficient and cost-effective ML classification approach to distinguish between different phases of thermoelectric materials can provide essential insights. Such an approach would complement existing regression models by offering valuable guidance for the design and discovery of high-performance thermoelectric materials.

To address this problem, we focus on distinguishing the phases of various thermoelectric materials in this study. We construct databases to identify the phase formations of different groups of thermoelectric materials. These groups include Half-Heusler (HH) compounds, which form an FCC structure; $Mg_{2}$(Si or Sb)-based alloys, which form a hexagonal structure; $Bi_{2}$$Te_{3}$-based alloys, which form a rhombohedral structure; transition metal (TM) chalcogenides, which generally exhibit a hexagonal structure; and (Pb, Sn, Ge) chalcogenides, which may adopt hexagonal, rhombohedral, or cubic structures. For oxide-based thermoelectric materials, we classify them into four structural categories: hexagonal, perovskite, orthorhombic, and rhombohedral. Using a Support Vector Machine (SVM) with previously developed alloy parameters [33], we classify various phases of thermoelectric materials. To further enhance model accuracy, we create a new set of raw features by incorporating additional elemental parameters alongside the alloy parameters. Moreover, we evaluate the model’s ability to distinguish between different phases through cross-validation across multiple thermoelectric material databases. The results demonstrate the model’s effectiveness in classifying thermoelectric phases that exhibit specific thermoelectric properties. Thus, the model developed herein provides an important grounding for understanding the structure–property relationships essential in the development of future thermoelectric materials.

## 2. Methods

**Figure 1:**
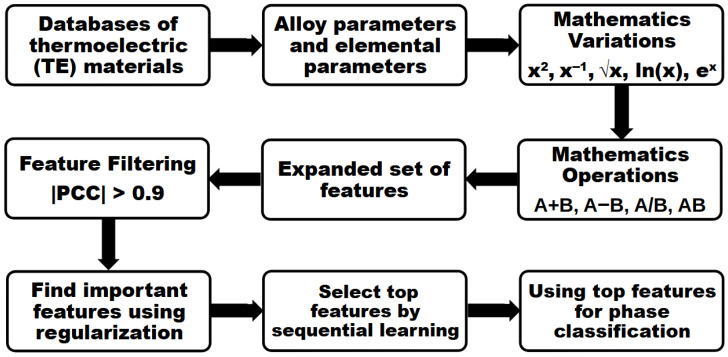
A flowchart is provided to illustrate the overall process of the method used in this work. The process begins with databases of thermoelectric materials, from which alloy parameters and elemental parameters are generated as raw features based on the alloys’ compositions. For feature engineering, mathematical variations and subsequent operations are applied to create an expanded set of features. To filter these features, the Pearson Correlation Coefficient (PCC) is used as a criterion: one feature is removed from any pair with an absolute PCC value greater than 0.9 (|PCC| > 0.9). After filtering, L1 regularization is applied to identify and select important features. Finally, best features are obtained from the sequential learning algorithm by minimizing the average prediction error, which are then used to train the phase classification model.

In this work, we adopt the ML phase classification models of Qi et al. with appropriate modifications [33] to classify thermoelectric crystal phases. The overall process of this method is illustrated in the flowchart shown in Figure 1. TE materials are grouped into databases based on their material classes and phases. These include Half-Heusler (HH), $Mg_{2}$(Si or Sb)-based alloys, $Bi_{2}$$Te_{3}$-based alloys, TM chalcogenides, (Pb, Sn, Ge) chalcogenides, and various oxides. Their respective crystal structures are as follows: HHs are FCC; $Mg_{2}$(Si or Sb)-based alloys are hexagonal; $Bi_{2}$$Te_{3}$-based alloys are rhombohedral, which consists of ${\left(Bi,Sb\right)}_{2}$$Te_{3}$, $Bi_{2}$${\left(Te,Se\right)}_{3}$, and doped derivatives of $Bi_{2}$$Te_{3}$ [12]; TM chalcogenides are hexagonal; (Pb, Sn, Ge) chalcogenides are either rhombohedral or cubic; and various oxides contain hexagonal, perovskite, or orthorhombic structures. Each model is trained independently on a specific phase-type database, such as the HH database. In this framework, an alloy known to form the HH phase is labeled as not forming the other phases. As a result, each model can only predict whether a given composition will form the specific phase it was trained on. For example, a model trained on the HH database will predict whether a composition can form the HH phase, regardless of whether the same composition is predicted to form (or not form) other phases by models trained on different databases. For oxides, the model can only distinguish whether a given composition forms a hexagonal phase, but not between different hexagonal phases. Random forest (RF) and Support Vector Machine (SVM) classification models are trained to categorize the phase formations of TE alloys. Only the results of SVM classification models are shown herein, because SVM models show mostly higher accuracy than RF models. The accuracy used to evaluate the models’ performance is the overall accuracy. For detailed comparisons between different ML classification models, refer to previous work by Qi et al. [33]. During each training, 80% of the data is used for training and the other 20% is for validations. This process is repeated ten times.

For feature selection, we start with raw features obtained from various thermodynamic and Hume-Rothery parameters [33]. These features are mixing entropy Δ$S_{mix}$, mixing enthalpy Δ$H_{mix}$(obtained from Miedama’s model) [48], Ω, Φ, η, $k_{1}^{cr}$, radius mismatch δ, $E_{2}$/$E_{0}$, electronegativity mismatch Δχ, and mean valence electron concentration VEC. The definitions of these raw features are listed in Table 1. We also utilize elemental parameters, obtained from Matminer [49], as raw features. These elemental parameters include covalent radius, first ionization energy, and Mendeleev number. For a given alloy, the elemental features also include the minimum, maximum, weighted average, standard deviation of the mean, and range of each elemental parameter. In total, 10 alloy parameters and 15 elemental parameters serve as the raw features in this work.

Feature engineering is employed to improve the performance of the machine learning model [33]. First, a new set of features is constructed from raw features X, using mathematical variations $x^{2}$, $x^{-1}$, $\sqrt{x}$, ln(x), and $e^{x}$. Then, the set of features is further expanded by grouping two mathematical variances, A and B, using the following arithmetic operations: A+B, A-B, A/B, and AB. To filter the expanded set of features, the Pearson Correlation Coefficient (PCC) is employed. For any feature pair with |PCC| > 0.9, only one is kept in the model. By doing this, any feature pairs that are strongly correlated, both positive and negative, are filtered down to one. Then, a logistic regression with L1 (or Lasso) regularization is used to directly select important features and eliminate useless features. This selection is achieved by minimizing the total prediction penalty, which is a trade-off between reducing prediction error and regulating the number of selected features. Finally, a sequential learning algorithm selects the best features by minimizing the average prediction error from thirty rounds of five-fold cross-validation. For each round, the top feature is selected for ML. After these steps, only the top features are used for phase classifications in this work. For alloy parameters, the top five features are $\Delta H_{mix}^{2}$, $VEC^{2}$, $\frac{\delta }{\Phi }$, Δχ, and $\frac{\Delta S_{mix}}{\eta }$. With the addition of elemental parameters, the top five features are $\Delta H_{mix}^{2}$, $\frac{covalent\;radius(weighted\;average)}{\Delta S_{mix}}$, $\Delta \chi^{2}$ x Mendeleev number (weighted average), $VEC^{2}$, and $\frac{\delta }{\Phi }$.

## 3. Results and Discussion

First, we examine the accuracy of phase classifications using alloy parameters as raw features. As shown in Table 2, using SVM, the accuracy ranges from 77% to 91% across material groups. In comparison, RF yields accuracy ranging from 72% to 87%, which justifies the selection of SVM for this study. The listed accuracy and the range of accuracy represent the average prediction accuracy and the range from ten repeated calculations with different random seeds, respectively, which play a role in feature selection, feature engineering, and the ML classification algorithm. The highest accuracy is for the prediction HH phase, with an accuracy of 91%. Then, there is a rather noticeable drop in accuracy to 84% to 85% for predicting $Mg_{2}$(Si or Sb)-based alloys, $Bi_{2}$$Te_{3}$-based alloys, and TM chalcogenides. A further drop in accuracy to 81% is seen in predicting (Pb, Sn, Ge) chalcogenides. The lowest accuracy is for predicting the oxides, which has 78% accuracy for perovskites and the orthorhombic phase, and 77% accuracy for the hexagonal and rhombohedral phases. We also examine specific examples where the model performs well and where it is less successful. For example, for HH compounds, the model accurately predicts the formation of the HH phase for TiCoSn and various doped alloys of TiCoSn, such as $TiFe_{0.1}Co_{0.9}Sb$ and $TiFe_{0.5}Co_{0.5}Sb$. However, the model fails to predict the HH phase formation for $ZrNiPb_{0.98}Bi_{0.02}$. From these results, the model can predict the inter-metallic HH phase with the highest accuracy. However, when the alloy group contains more small-group (non-metallic) elements, such as Si and Te, the accuracy decreases to around 85%. Further decreases are seen in the oxides, which contain non-metal O. A possible explanation for this decrease in accuracy is the incomplete alloy parameters for some of these semi-metal or non-metal alloys. While parameters, such as entropy S, are well-defined for any given alloy, other parameters, including mixing enthalpy ΔH, are estimations, which can be inaccurate. Furthermore, parameters, such as melting temperature $T_{m}$, can vary greatly for different i-j element pairs. Another plausible reason is that HH compounds form a well-defined FCC structure. In contrast, other material groups, such as chalcogenides, can adopt multiple crystal structures, include hexagonal, rhombohedral, or cubic, making their phase identification more challenging. Despite these limitations, the model is still able to predict the phase formation with a reasonable prediction accuracy of 77% or above.

Then, we include several elemental parameters, including covalent radius, first ionization energy, and Mendeleev number, to re-examine the model. By incorporating elemental parameters with alloy parameters, the prediction accuracy increases by 1% to 4% for all material groups, as shown in Table 3. Starting with the HH group, the prediction accuracy increases from 91% to 92%. For $Mg_{2}$(Si or Sb)-based alloys, the prediction accuracy increases from 84% to 86%. For $Bi_{2}$$Te_{3}$-based alloys, the prediction accuracy increases from 85% to 86%. For TM chalcogenide alloys, the prediction accuracy increases from 84% to 86%. For (Pb, Ge, or Sn) chalcogenide alloys, the prediction accuracy increases from 80% to 82%. For the oxides, the hexagonal phase increases from 77% to 80%, the perovskite phase increases from 78% to 81%, the orthorhombic phase increases from 78% to 80%, and the rhombohedral phase increases from 77% to 81%. These increases in prediction accuracy can be attributed to the inclusion of more well-defined elemental parameters in the model and the incorporation of some missing physical concepts in alloy parameters. These physical concepts, such as covalent radius and first ionization energy, can play a key role in phase formation. Thus, including these parameters can improve the model. For HH, since the original model with alloy parameters can already predict well with well-defined alloy parameters, the increase in prediction accuracy is marginal, from 91% to 92%. More notable improvements of 3% to 4% are found in the oxide group, which originally contained more estimated parameters. Thus, incorporating elemental parameters has a greater influence on the oxide group. Overall, by using alloy and elemental parameters with feature engineering, the model achieves prediction accuracies of 80% or higher across the nine different material groups.

To examine the model’s ability to distinguish between material groups, we use cross-validation to check the model. In this cross-validation, each target material group is tested using models trained on a different material dataset. Table 4 shows the results of this cross-validation. As shown in Table 4, the diagonal terms are the accuracy of the targeted material group trained using the respective dataset. These accuracies are the same as those obtained in Table 2, because they are the same model. The off-diagonal terms are the false positive rate of targeted materials trained using a different dataset. In other words, it represents the percentage of alloys from another material group that a given model falsely predicts as belonging to the trained material group. For example, in the HH column and $Mg_{2}$(Si, Sb)-based row, the false positive rate is 0.05. This means that when we use a model trained with HH datasets, and let the model predict if alloys are from the $Mg_{2}$(Si, Sb)-based dataset, the model falsely predicts 5% of those materials will form the HH phase. Looking at Table 4, for the majority of these cross-validations, the false positive rate ranges from 0.01 to 0.10. The material groups that these models have trouble distinguishing are between the TM chalcogenides and (Pb, Ge, or Sn) chalcogenides, where the false positive rate reaches 0.21 and 0.28 using the model trained by (Pb, Ge, or Sn) chalcogenides to predict TM chalcogenides and using the model trained by TM chalcogenides to predict (Pb, Ge, or Sn) chalcogenides, respectively. This is likely due to the fact that they are overlapped in the material phase space between these two material groups, as both contain chalcogen elements (S, Se, or Te). In addition, both chalcogenide groups share similar crystal structures; as mentioned earlier, both can adopt hexagonal, rhombohedral, or cubic structures, which may also contribute to the model’s confusion between these two groups. For oxides, when presented with an oxide, the model attempts to classify it into one of the four oxide phase types included in this study: hexagonal, perovskites, orthorhombic, or rhombohedral. Ideally, the model would be able to accurately differentiate among all four categories. However, as shown in Table 2 and Table 3, the overall prediction accuracy for oxides ranges from 77% to 81%. As a result, there are instances of false positives, where the model predicts the formation of multiple oxide phases for a single material. Addressing this limitation will require future experimental validation and more precise ab initio calculations to obtain detailed alloy-specific parameters, which could enhance the model’s predictive accuracy. From this cross-validation, the models show robustness in identifying and distinguishing different TE phases.

## 4. Conclusions

We have employed Support Vector Machine (SVM) to predict phases of thermoelectric (TE) alloys, with the goal of identifying and distinguishing different TE phases so that specific phases can be predicted correctly. Our initial model, using only alloy parameters, achieved accuracies ranging from 77% to 91%. With the incorporation of additional elemental parameters, the accuracies improved to between 80% and 92%. To further evaluate the model’s robustness, we performed cross-validation across various TE material groups. Notably, the model achieved a low false positive rate of 0.01 when predicting whether chalcogenide or oxide alloys would incorrectly form the HH phase, and vice versa. However, the model struggled to distinguish between transition metal (TM) chalcogenides and (Pb, Ge, or Sn)-based chalcogenides, with false positive rates reaching 0.21 and 0.28, respectively. With future experimental validation and more accurate ab initio calculations, the precision of alloy parameters can be significantly improved, which is expected to enhance the model’s performance. Overall, this study provides an important step toward the reliable identification of phase formations in TE alloys, which serve as a critical foundation for the design and discovery of high-performance TE materials for future energy applications.

## Figures and Tables

**Table 1:** Definitions of alloy parameters used as raw features in this work.

Definition	Comments
$\Delta S_{mix}=-R\sum_{i=1}^{N}c_{i}ln\left(c_{i}\right)$	R = the gas constant
$\Delta H_{mix}=\sum_{i=1,i\neq j}^{N}4\Delta H_{mix}^{ij}c_{i}c_{j}$	$c_{i}$ = the atomic percentage of the i-th element for an N-element alloy
	$\Delta H_{i,j}^{mix}$ = the binary mixing enthalpy obtained from Miedama’s model [48] of i-j elemental pair
$\Omega =\frac{T_{m}\Delta S_{mix}}{|\Delta H_{mix}|}$	$T_{m}$ = alloy melting temperature
$\Phi =\frac{\Delta G_{SS}}{-|\Delta G_{max}|}$	$\Delta G_{SS}$ = the Gibbs free energy change for forming a fully disordered solid solution phase
$\eta =\frac{-T_{ann}\Delta S_{mix}}{|\Delta H_{f}|}$	$\Delta G_{max}$ = the largest absolute Gibbs free energy for forming the strongest binary compound
	$T_{ann}$ = annealing temperature, or if $T_{ann}$ is unknown, $T_{ann}$ = 0.8 $T_{m}$
$k_{l}^{cr}=\frac{1-\frac{0.4T_{m}\Delta S_{mix}}{\Delta H_{mix}}}{\frac{\Delta H_{IM}}{\Delta H_{mix}}}$	$T_{m}=\frac{\sum_{i\neq j}T_{i-j}c_{i}c_{j}}{\sum_{i\neq j}c_{i}c_{j}}$ where $T_{i-j}$ is the melting temperature of the i-j elements
	$\Delta H_{f}$ = the most negative binary mixing enthalpy for forming inter-metallics
$\delta =\sqrt{\sum_{i=1}^{N}c_{i}{\left(1-\frac{r_{i}}{\sum_{j=1}^{N}c_{j}r_{j}}\right)}^{2}}$	$\Delta H_{IM}$ = mixing enthalpy for forming inter-metallics
$\frac{E_{2}}{E_{0}}=\frac{\sum_{j\geq i}^{N}c_{i}c_{j}\left|r_{i}+r_{j}-2{\bar{r}}^{2}\right|}{{\left(2\bar{r}\right)}^{2}}$	$r_{i}$ = the atomic radius of the i-th element
	$\bar{r}=\sum_{i=1}^{N}c_{i}r_{i}$ = average atomic radius
$\Delta \chi =\sqrt{\sum_{i=1}^{N}c_{i}{\left(\chi_{i}-\sum_{j=1}^{N}c_{j}\chi_{j}\right)}^{2}}$	$\chi_{i}$ = electronegativity of i-th element
$VEC=\sum_{i=1}^{N}c_{i}VEC_{i}$	$VEC_{i}$ = valence electron count of the i-th element

**Table 2:** Results of phase classifications using alloy parameters with feature engineering. Accuracy is listed for each material group dataset, with the highest accuracy for HH at 91%, and the lowest accuracy for hexagonal and rhombohedral oxides at 77%. The range of accuracy from ten repeated calculations is in parentheses.

Materials Group	Accuracy
HH	91% (85–96%)
$Mg_{2}$ + (Si or Sb)-based	84% (78–90%)
BiTe-based	85% (80–88%)
TM Chalcogenides	84% (79–88%)
(Pb, Ge, or Sn) Chalcogenides	80% (76–84%)
Oxides (Hexagonal)	77% (74–84%)
Oxides (Perovskites)	78% (75–82%)
Oxides (Orthorhombic)	78% (73–81%)
Oxides (Rhombohedral)	77% (71–84%)

**Table 3:** Results of phase classification using alloy and elemental parameters with feature engineering. Accuracy is reported for each material group dataset, with the highest accuracy observed for Half-Heusler (HH) compounds at 92%, and the lowest for hexagonal and orthorhombic oxides at 80%. The range of accuracy from ten repeated calculations is shown in parentheses.

Materials Group	Accuracy
HH	92% (85–96%)
$Mg_{2}$ + (Si or Sb)-based	86% (81–90%)
BiTe-based	86% (81–91%)
TM Chalcogenides	86% (81–90%)
(Pb, Ge, or Sn) Chalcogenides	82% (77–86%)
Oxides (Hexagonal)	80% (76–85%)
Oxides (Perovskites)	81% (77–82%)
Oxides (Orthorhombic)	80% (76–83%)
Oxides (Rhombohedral)	81% (77–84%)

**Table 4:** Cross-validation table between materials from different datasets. Diagonal components (red) are the accuracy of targeted materials trained using respective datasets, which is the same as the accuracy shown in Table 2. Off-diagonal components (blue and black) are the false positive rates of targeted materials trained using a different dataset. As indicated by the right and down arrows, each column represents a distinct training set, and each row corresponds to a specific target alloy group used for validation. Besides the TM chalcogenides and (Pb, Ge, or Sn) chalcogenides (blue), which have 0.21 and 0.28 false positives, others show false positives of 0.10 or less.

Training Sets → Targeted Alloys ↓	HH	${\mathrm{Mg}}_{2}$ + (Si or Sb)-Based	BiTe-Based	TM Chalcogenides	(Pb, Ge, or Sn) Chalcogenides	Oxides
**HH**	0.91	0.06	0.03	0.10	0.04	0.02
**${\mathrm{Mg}}_{2}$ + (Si or Sb)-based**	0.05	0.84	0.03	0.08	0.02	0.02
**BiTe-based**	0.01	0.02	0.85	0.01	0.02	0.01
**TM Chalcogenides**	0.07	0.05	0.02	0.84	0.21	0.02
**(Pb, Ge, or Sn) Chalcogenides**	0.01	0.01	0.02	0.28	0.80	0.01
**Oxides**	0.01	0.01	0.01	0.01	0.01	0.77

## Data Availability

The original data presented in the study are openly available in FigShare at https://doi.org/10.6084/m9.figshare.30041518.v1. accessed on 1 September 2025.
